# Good manufacturing practice inspections conducted by Tanzania medicines and medical devices authority: a comparative study of two fiscal years from 2018 to 2020

**DOI:** 10.1080/20523211.2024.2399722

**Published:** 2024-09-16

**Authors:** Raphael Zozimus Sangeda, Chimpaye Julius Ndabatinya, Maganga Bundala Maganga, Emmanuel Alphonce Nkiligi, Yonah Hebron Mwalwisi, Adam Mitangu Fimbo

**Affiliations:** a Department of Pharmaceutical Microbiology, Muhimbili University of Health and Allied Sciences, Dar es Salaam, Tanzania; b Tanzania Medicines and Medical Devices Authority, Dodoma, Tanzania

**Keywords:** Good manufacturing practice (GMP) inspections, Tanzania Medicines and Medical Devices Authority (TMDA), quality, safety, and efficacy, regulation of medicines, assessment and registration of medicinal products, post-market surveillance

## Abstract

**Background:**

Good Manufacturing Practices (GMP) is the bedrock of quality assurance in the pharmaceutical industry that ensures that products are consistently produced and controlled according to quality standards. This study compared the GMP conformance of pharmaceutical facilities across two fiscal years, 2018/2019 and 2019/2020, using the East African GMP Compendium on Good Manufacturing Practices, 2014, as a benchmark.

**Methods:**

We analyzed the proportion of conformance of foreign pharmaceutical industries to GMP standards and reported the aggregated data over a two-year period.

**Results:**

Inspected facilities had notable non-conformances, most commonly related to laboratory quality control and premises. We noted a downward trend in conformance in 2019/2020 compared with 2018/2019, with only 32.9% of facilities adhering to EAC GMP requirements, down from 50% in the previous year. The COVID-19 pandemic has affected the ability to conduct on-site inspections, and may have contributed to the lower conformance rate.

**Conclusions:**

These findings underscore the crucial need to continue GMP inspections and the importance of taking corrective actions to ensure adherence to the quality standards for products marketed in Tanzania. The study further revealed the significance of desk reviews in assisting regulatory authorities in facing unforeseen challenges such as pandemics.

## Background

Ensuring the quality, safety, and efficacy of medicine is a fundamental priority in modern healthcare. With the increased reliance on pharmaceutical technology, there is an imperative need for robust regulatory systems to supervise and control the production and distribution of medical products. In East Africa, the Tanzania Medicines and Medical Devices Authority (TMDA) is a reputable regulatory authority enforcing this role. As an Executive Agency under the Ministry of Health, TMDA shoulders significant public health mandates, mainly regulating and controlling the quality, safety, and efficacy of medicines, medical devices, diagnostics, and tobacco products (Tanzania Medicines and Medical Devices Authority (TMDA), n.d.-d). One of the core functions of TMDA is to inspect manufacturing facilities to ensure conformance with Good Manufacturing Practice (GMP) standards and regulations (Tanzania Medicines and Medical Devices Authority (TMDA), n.d.-b, n.d.-c).

TMDA safeguards public health by regulating medicines (human, veterinary, and herbal) and medical devices and ensuring their quality, safety, and efficacy. This is achieved through a rigorous product registration process, whereby medical products are evaluated and corresponding manufacturing facilities are inspected for conformance with the East African GMP Compendium on Good Manufacturing Practices, 2014. This requirement is pursuant to Section 51 of the Tanzania Medicines and Medical Devices Act, Cap 219 (The United Republic of Tanzania, n.d.). Inspections are guided through a risk-based approach, as delineated in the standard operating procedure (SOP) number TMDA/DMC/MCIE/SOP/007, to prepare GMP inspections of pharmaceutical manufacturing facilities (Tanzania Medicines and Medical Devices Authority (TMDA), n.d.-b, n.d.-c). This approach aligns with global best practices in regulatory oversight. It is similarly utilised by other National Drug Regulatory Authorities (NDRAs) in various jurisdictions, such as the US Food and Drug Administration (FDA) and the European Medicines Agency ((EMA), n.d.; FDA, n.d.-a).

The registration process of the pharmaceutical regulatory ecosystem and corresponding GMP inspections are critical. They form the backbone of regulatory mandates to ensure the production of high-quality, safe, and effective pharmaceutical products for public benefit. However, few studies (Arik et al., 2020; Ndomondo-Sigonda et al., 2021; Ocan et al., 2023) have highlighted gaps in conformance with GMP standards in the East African community, highlighting the need for more comprehensive oversight and regulatory control. These deficiencies mainly concern regulatory tools to effectively regulate medicines with minimal resources available. Furthermore, several studies have detected discrepancies in the quality of pharmaceutical products across the East African region, emphasising the need to strengthen the harmonisation of regulatory systems (Mackintosh et al., 2013; Mujinja et al., 2014). There is also limited research that compares GMP inspection practices and outcomes across different countries in East Africa. Given the increasing importance of regional harmonisation in pharmaceutical regulations, such comparisons can provide valuable insights (Mackintosh et al., 2013). This is particularly pivotal given the recent move towards mutual recognition of GMP inspection outcomes among the East African Community Partner States (Mashingia et al., 2023).

Despite the importance of GMP inspections for the quality of imported medicines, there is a paucity of studies reporting the results of GMP inspections in manufacturing facilities. Therefore, in this study, we reviewed the conformance to GMP of pharmaceutical industries inspected by TMDA over two years and compared their conformance rates. This study contributes to our understanding of the challenges and opportunities in pharmaceutical regulation in East Africa and the need to strengthen regional and global regulatory systems. By disclosing areas of deficiencies, we encourage the industry to improve documentation and abide by country-specific regulations regarding GMP compliance and ultimately, the availability of high-quality pharmaceutical products. The study may also shed light on other regulatory authorities in the East African region in light of the ongoing harmonisation of medical product regulation. It may highlight areas of standard training and provide opportunities for joint inspection (Ndomondo-Sigonda et al., 2021).

## Methods

### Study settings

This study was conducted across a range of manufacturing facilities operating under the regulatory oversight of the Tanzania Medicines and Medical Devices Authority (TMDA), located in several countries around the world, during the 2018/2019 and 2019/2020 fiscal years. These inspections focused on overseas pharmaceutical manufacturers exporting medicines to Tanzania, including India, China, Kenya, Jordan, Egypt, Turkey, Bangladesh, Pakistan, Indonesia, Palestine, Vietnam, Malaysia, Oman, Singapore, South Africa, Thailand, Nepal, Uganda, and the United Arab Emirates.

### Composition of inspectors

The inspections involved 40 inspectors who formed 20 teams (2019/2020) to inspect the facilities in Africa, Asia, and Europe. The teams comprised a lead inspector and a co-inspector appointed by the Director General as per the SOP for the preparation of GMP inspections. Inspection reports, including observations and inspection findings, were peer-reviewed by the TMDA GMP Technical Committee. The inspection teams presented their findings for consideration before approval and dispatch of inspection reports to the applicants.

### Assessment tool

To assess the conformance of medicine manufacturing facilities, we used the Compendium of GMP Technical Documents for Harmonisation of Medicines Regulation in the East African Community 2014 (Tanzania Medicines and Medical Devices Authority (TMDA), n.d.-a). This Compendium served as a definitive reference point, providing a comprehensive guide to GMP and offering a shared benchmark for regulatory harmonisation in East Africa. The Compendium contains technical documents outlining the guidelines, standards, and expectations of GMP in the pharmaceutical industry. These documents encompass various areas, including the manufacturing process, quality assurance, premises, equipment, personnel, and documentation, noting any deviations from these standards identified as non-conformance. Using this Compendium, the findings were grouped, allowing for an accurate, objective, and comparative assessment of quality in the inspected sites.

The principles outlined in the EAC GMP Compendium (2014) were particularly pertinent to the observed non-conformances during the study period. These principles include Pharmaceutical Quality Systems, Premises, Personnel, and Heating Ventilation and Air Conditioning (HVAC) systems. In addition, the quality of water-treated plants was assessed. Quality control measures and the state of equipment and production processes were also evaluated. Finally, documentation, which is a critical component of GMP, was scrutinised to ensure completeness and adherence to GMP norms.

### Good manufacturing practices (GMP) assessment

This study conducted an exhaustive and systematic analysis of the GMP inspection reports. These reports were sourced from 196 facilities inspected by TMDA during the 2018/2019 and 2019/2020 fiscal years. The attributes assessed included the state of the facilities, the type of products manufactured (human or veterinary), and the type of inspection conducted. The figures were computed as the number of facilities and the corresponding percentages of the total number of facilities inspected in each period. Three types of inspection were conducted: pre-registration inspection, re-inspection, and renewal inspection. A pre-registration inspection aims to verify whether a product or system meets specified requirements. A regulatory authority performs a pre-registration inspection before a product enters the market. In pharmaceutical facilities, this involves checking whether the facility meets standards for manufacturing processes, personnel qualifications, equipment quality, and record-keeping, among others. Re-inspection involves re-evaluating a previously inspected facility that has observed critical non-conformances. Re-inspections are usually conducted for facilities that do not comply with stipulated GMP standards during the initial GMP inspection. However, renewal-type GMP inspection constitutes an inspection conducted at a previously compliant facility where the validity of conformance has expired. To ensure that the facility meets the GMP standards, it should be renewed through a renewal GMP inspection process.

### Facility conformance status

During GMP inspections, non-conformances are categorised into three distinct classes: minor, major, and critical. Each category is defined by the potential impact of non-conformance on product quality and safety. Minor non-conformances are those with a minimal likelihood of affecting product quality. These non-conformances do not significantly impact the product or pose a risk to consumers. On the other hand, major non-conformances potentially reduce a product's quality without posing a direct threat to the consumer. These non-conformances do not influence the product's strength, identity, purity, or safety but might affect the overall user experience. Critical non-conformances present the most significant concern, as they pose a direct health concern to consumers. These factors substantially impact the strength, identity, purity, and safety of a product.

To elucidate the degree of conformance across the inspected facilities, we calculated the number and percentage of facilities with non-conformance in each category. In addition, we performed a detailed critical examination of non-conformances against the GMP principles defined in the EAC GMP compendium (Tanzania Medicines and Medical Devices Authority (TMDA), n.d.-a). These principles are the gold standards for pharmaceutical manufacturing.

Our analysis, showing the frequency and nature of non-conformances, was then organised and visualised using tables and graphs.

The final inspection status indicated that the facilities complied with the standards. In addition, some were marked ‘On Demand,’ meaning that further scrutiny of the facility was required. The ‘Complied’ category represents those facilities that fully met the required standards during the inspection.

The ‘on-demand’ category indicates those facilities that might have had major non-conformances that would require rectification and may also require a follow-up or more detailed inspection. The ‘Not Complied’ category represented facilities that did not meet the standards and exhibited significant non-conformance during inspection.

### Data analysis

The collected quantitative data were analyzed using SPSS software version 26.0. Data were cleaned to check for consistency and accuracy. Descriptive statistics were used to summarise the data, and categorical variables were presented as frequency distributions with percentages, tables, or graphs.

### Ethical considerations

All principles of subject privacy and confidentiality were observed throughout the study. The names of the participants and the industries involved were anonymous in this study to conceal their identities. Permission to publish this work was obtained from the Tanzania National Health Research Ethics Committee (NatHREC).

## Results

A total of one hundred and ninety-six inspections were conducted, 120 and 76 in fiscal years 2019/2020 and 2018/2019, respectively (Table 1).

**Table 1.** General attributes of facilities inspected over two periods (2018/2019 and 2019/2020), including the type of production, the type of inspection conducted, the country, and the final outcome of the inspection.

Attributes	Fiscal year				Overall total for both periods	
	2018/2019		2019/2020			
	*N* = 120	%	*N* = 76	%	*N* = 196	%
**Year**						
2018	70	58.3		0.0	70	35.7
2019	50	41.7	65	85.5	115	58.7
2020			11	14.5	11	5.6
**Human or veterinary product**						
Human	111	92.5	68	89.5	179	91.3
Veterinary	9	7.5	8	10.5	17	8.7
**Inspection type**						
Pre-registration Inspection	60	50.0	39	51.3	99	50.5
Re-Inspection	10	8.3	8	10.5	18	9.2
Renewal GMP Inspection	50	41.7	29	38.2	79	40.3
**Final inspection status**						
Complied	60	50.0	25	32.9	85	43.4
On-Demand	35	29.2	43	56.6	78	39.8
Not Complied	25	20.8	8	10.5	33	16.8
**Country**						
India	82	68.3%	46	60.5%	128	65.3%
China	9	7.5%	4	5.3%	13	6.6%
Kenya	7	5.8%	2	2.6%	9	4.6%
Jordan	4	3.3%	2	2.6%	6	3.1%
Egypt	3	2.5%	2	2.6%	5	2.6%
Turkey	4	3.3%	1	1.3%	5	2.6%
Bangladesh	0	0.0%	4	5.3%	4	2.0%
Pakistan	0	0.0%	4	5.3%	4	2.0%
Indonesia	1	0.8%	2	2.6%	3	1.5%
Palestine	1	0.8%	2	2.6%	3	1.5%
Vietnam	0	0.0%	3	3.9%	3	1.5%
Malaysia	2	1.7%	0	0.0%	2	1.0%
Oman	2	1.7%	0	0.0%	2	1.0%
Singapore	0	0.0%	2	2.6%	2	1.0%
South Africa	0	0.0%	2	2.6%	2	1.0%
Thailand	2	1.7%	0	0.0%	2	1.0%
Nepal	1	0.8%	0	0.0%	1	0.5%
Uganda	1	0.8%	0	0.0%	1	0.5%
United Arab Emirates	1	0.8%	0	0.0%	1	0.5%

In the year category, it can be observed that in the first period (2018/2019), 70 facilities (58.3%) were inspected in 2018, whereas 50 facilities (41.7%) were inspected in 2019. In the second period (2019/2020), inspections were conducted only in 2019 and 2020, with 65 facilities (85.5%) inspected in 2019 and 11 facilities (14.5%) inspected in 2020 (Table 1).

Regarding the type of product, most facilities were involved in the production of human medicines in both periods: 111 (92.5%) in the first period and 68 (89.5%) in the second period. A smaller proportion was involved in veterinary production: 9 facilities (7.5%) in the first period and eight facilities (10.5%) in the second period (Table 1).

Pre-registration Inspection was the most common type in both periods, accounting for 50% of inspections in the first period (60 facilities) and 51.3% in the second period (39 facilities). Re-inspections were the most common type in both periods: 10 facilities (8.3%) in the first period and eight facilities (10.5%) in the second period. Renewal GMP inspections accounted for 41.7% (50 facilities) in the first period and 38.2% (29 facilities) in the second period.

India had the highest number of facilities inspected in both periods, with 82 facilities (68.3%) in the first period and 46 (60.5%) in the second period, resulting in a combined total of 128 facilities (65.3%).

China had the second highest number of facilities, with nine facilities (7.5%) in the first period and four facilities (5.3%) in the second period, making a combined total of 13 facilities (6.6%).

Kenya ranked third, with seven facilities (5.8%) in the first period and two (2.6%) in the second period, with a combined total of nine facilities (4.6%). Other countries, including Jordan, Egypt, Turkey, Bangladesh, Pakistan, Indonesia, Palestine, Vietnam, Malaysia, Oman, Singapore, South Africa, Thailand, Nepal, Uganda, and the United Arab Emirates, accounted for the remaining facilities inspected.

In the first period (2018/2019), 60 facilities (50.0%) complied, whereas in the second period (2019/2020), 25 facilities (32.9%) complied, resulting in a total of 85 facilities (43.4%) across both periods.

During the first period, 35 facilities (29.2%) fell into the ‘On Demand’ category, while in the second period, this number increased to 43 facilities (56.6%), making a total of 78 facilities (39.8%) across both periods.

In the first period, non-conformance was noted in 25 facilities (20.8%), whereas in the second period, eight facilities (10.5%) did not comply. Thus, 33 facilities (16.8%) did not comply with either period.

**Figure 1.**
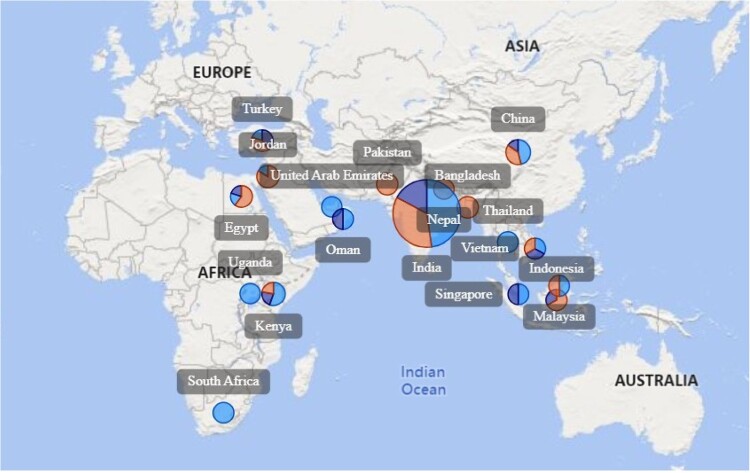
Geopolitical locations of inspected facilities across the globe.

The inspections covered the geopolitical zones in Africa, Asia, and Europe (Figure 1).

**Figure 2.**
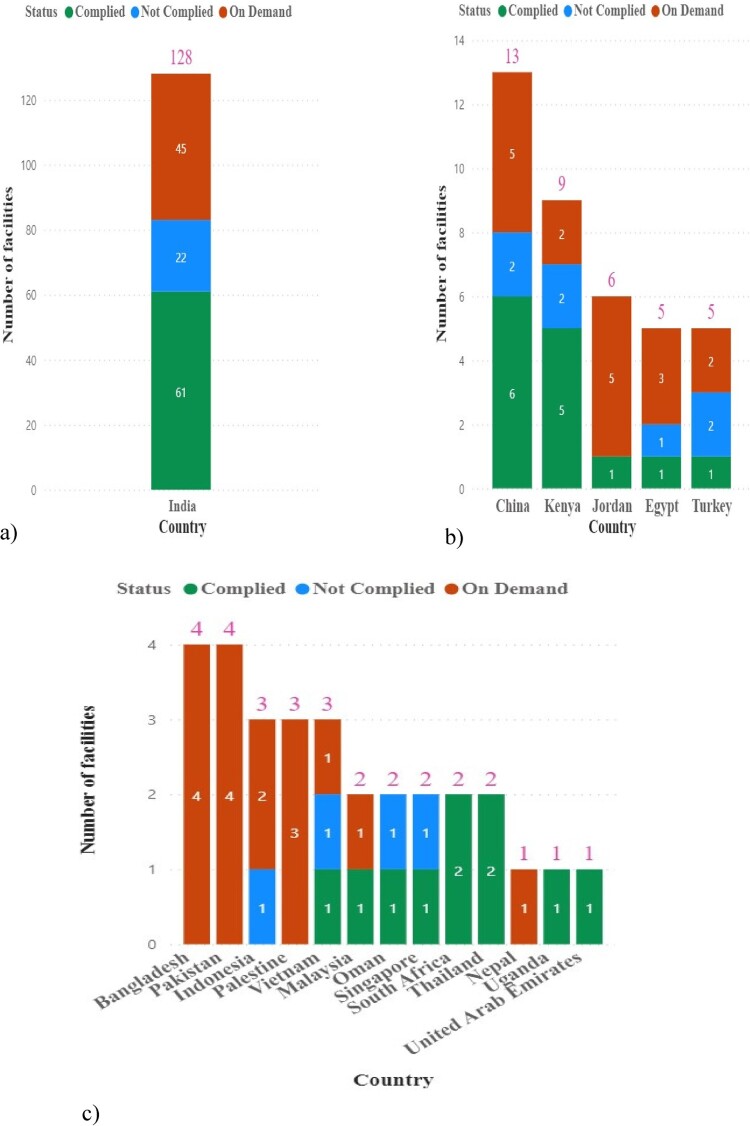
Status of each country facility GMP inspection for countries with (a) more than 15 facilities inspected (b) more than four, and (c) less than or equal to four.

The status of inspections in each country indicates that India had the highest number of inspections conducted at 128, of which 61 complied, 45 were on demand, and 22 were given critical non-conformances and did not comply with the GMP (Figure 2).

**Figure 3.**
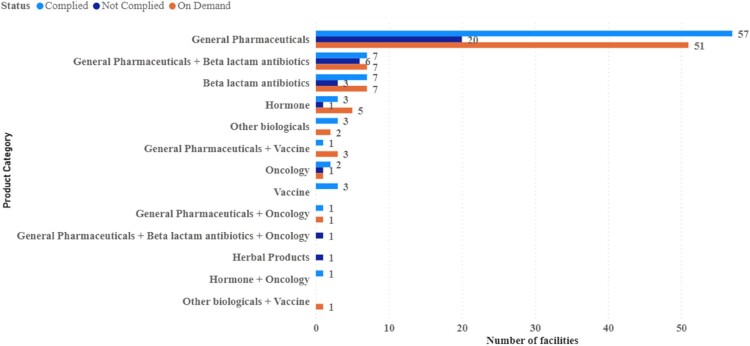
Status per product category.

Most inspected facilities were general pharmaceutical products (Figure 3): 57 complied, 51 on demand, and 20 did not comply.

The GMP principle with the most non-conformance in the two inspection periods was good practice in quality control (48.5%), followed by premises (45.5%) (Table 2).

**Table 2.** Percentage of non-conformance in different GMP systems/principles over two fiscal years.

GMP Systems/ Principles	Financial year					
	2018/2019		2019/2020		Both periods	
	Number of facilities	%^a^	Number of facilities	%^a^	Number of facilities	%^a^
Good Practices in Quality control	13	52.0%	3	37.5%	16	48.5%
Premises	12	48.0%	3	37.5%	15	45.5%
HVAC	10	40.0%	4	50.0%	14	42.4%
Good Practices in Production	6	24.0%	6	75.0%	12	36.4%
Validation	8	32.0%	0	0.0%	8	24.2%
Equipment	4	16.0%	3	37.5%	7	21.2%
Documentation	6	24.0%	0	0.0%	6	18.2%
Pharmaceutical Water Systems	4	16.0%	1	12.5%	5	15.2%
QMS	3	12.0%	1	12.5%	4	12.1%
Self-inspection	1	4.0%	1	12.5%	2	6.1%
Material Management	1	4.0%	0	0.0%	1	3.0%
Sanitation and Hygiene	1	4.0%	0	0.0%	1	3.0%
Total premises with non-critical conformance	25	100.0%	8	100.0%	33	100.0%

Key: HVAC: Heating Ventilation and Air Conditioning; QMS: Pharmaceutical Quality Management Systems.

^a^ The percentage calculation will not add 100 per financial period of inspections because a facility may have multiple critical non-conformities that fall under different principles.

This is a comparative analysis of critical non-conformances against various GMP principles across the two fiscal years, 2018/2019 and 2019/2020, on 33 facilities over both periods. Regarding good quality control practices, 16 facilities (48.5%) were found to have critical non-conformance. This was followed by premises, where 15 facilities (45.5%) showed non-conformance with the premises. The following principles were used: Heating, Ventilation, and Air Conditioning (HVAC), with 14 facilities (42.4%) displaying non-conformance. The principles with the lowest percentage of non-conformance were Material Management and Sanitation and Hygiene, both at one facility (3.0%) (Table 2).

The largest bump in non-conformance from 2018/2019 to 2019/2020 was in good practice in terms of production, which increased from six facilities (24.0%) to 12 facilities (36.4%). There was a significant decrease in non-conformance with the validation principle, which fell from eight facilities (32.0%) in 2018/2019 to zero (0%) in 2019/2020, indicating possible improvements or changes in this area.

## Discussion

This study reviewed the conformance to GMP of international pharmaceutical industries inspected by TMDA over two years and compared their conformance rates.

GMP inspections play a pivotal role in ensuring the quality, efficacy, and safety of pharmaceutical products, as illustrated by TMDA's rigorous inspection practices (World Health Organization., n.d.). Such practices ensure that only products manufactured from GMP-compliant facilities are registered and marketed in Tanzania, thereby safeguarding public health (Covarrubias et al., 2022). A significant number of inspected facilities in the two fiscal years were based in India (65.3%), followed by China (6.6%) and Kenya (4.6%). The geographical distribution of the inspected facilities reflects the status of these countries as prominent suppliers of pharmaceutical products in Tanzania. The observed pattern aligns with data suggesting that India is the leading exporter of pharmaceutical products globally, particularly to Africa, with China and Kenya also playing significant roles in the pharmaceutical supply chain (Bjerke, 2022; Conteh et al., 2022; Hamim et al., 2021; Sangeda, Baha, et al., 2021; Sangeda, Saburi, et al., 2021; Wande et al., 2019).

This strong dependence on imports underlines the need for stringent quality control measures to ensure the safety, quality, and efficacy of imported pharmaceuticals. Furthermore, it highlights the importance of fostering collaboration between TMDA and foreign NDRAs to harmonise regulatory processes to safeguard public health.

In the 2019/20 fiscal year, TMDA planned to inspect 135 pharmaceutical manufacturing facilities overseas. However, owing to travel restrictions from the COVID-19 pandemic (O’Brien et al., 2021), only 76 facilities were inspected, which is equivalent to 56.3% of the planned inspections. This situation highlights the disruptions caused by the pandemic to global regulatory activities, as other NDRAs have reported similar trends. For instance, the US FDA noted a significant decrease in inspections conducted in 2020 owing to COVID-19 travel restrictions (FDA, n.d.-b). Similarly, the EMA acknowledged that the pandemic posed tremendous challenges to GMP inspections ((EMA), n.d.). These global disruptions have underscored the need for innovative strategies, such as remote and hybrid inspections (O’Brien et al., 2021; Velásquez, 2022), which can be leveraged to ensure the continuity of this important regulatory function. In the face of these challenges, TMDA and other NDRAs have demonstrated remarkable resilience, conducting inspections amidst unprecedented circumstances and ensuring the continued availability of safe, effective, and quality medicinal products.

The lessons learned from this crisis have been vital in reshaping global regulatory strategies. The TMDA's approach to adapting its inspection procedures in response to the pandemic is a testament to this fact. In the 2020/2021, 2021/2022, and 2022/2023 fiscal years, the Authority opted for desk reviews as an alternative to physical site inspections. This shift was congruent with the recommendations of the World Health Organisation (WHO), which proposed desk reviews as an alternative strategy in times of travel restrictions and issued guidelines on performing the same (O’Brien et al., 2021; Velásquez, 2022). TMDA's adaptive measures also echo similar strategies implemented by other global NDRAs. For instance, the US FDA in the United States adopted a risk-based approach to prioritise domestic over foreign inspections while enhancing the use of alternative review tools during the pandemic (Diak et al., 2023; Mofid et al., 2021).

Similarly, the EMA noted an increased reliance on distant assessments during this period (Klein et al., 2022). While the shift to desk reviews ensured the continuity of regulatory oversight during the crisis, the TMDA resumed physical inspections in the second half of the fiscal year 2022/2023. Several studies have shown that physical inspections remain essential to the regulatory process owing to their thoroughness and the opportunity to interact directly with facility personnel [28–30]. Therefore, TMDA’s strategy demonstrates a commendable balance between adaptability in the face of crises and commitment to high standards of regulatory oversight. It is crucial for regulatory authorities worldwide to learn lessons to enhance their regulatory mandates.

The TMDA has adopted reliance mechanisms as a strategic response to the challenges posed by the pandemic and due to human resource shortages, notably through the implementation of desk reviews in 2020. This innovative approach facilitates the evaluation of manufacturing facilities based on documentation and previous inspections, thereby significantly reducing reliance on on-site inspections. TMDA has established specific guidelines for conducting these desk reviews, outlining the criteria for selecting facilities for this streamlined process. Eligible facilities were previously inspected by the Stringent Regulatory Authority (SRA) and the WHO (Miletic et al., 2023). Moreover, the TMDA has developed comprehensive guidelines for reliance, further supported by its participation in the WHO Collaborative Procedure and the implementation of an abridged assessment process. Collectively, these frameworks facilitate a streamlined regulatory approach, allowing TMDA to conduct assessments efficiently by recognising regulatory partners and international standards. These reliance mechanisms optimise the regulatory review process and ensure timely access to safe and effective medical products in Tanzania, demonstrating a commitment to global best practices in regulatory oversight.

Moreover, TMDA has been actively participating in the ZaZiBoNa Collaborative Procedure for Medicines Registration within the Southern African Development Community (SADC) region since March 2019 (Sithole et al., 2020). ZaZiBoNa is a pivotal work-sharing initiative that enhances collaborative medicine registration across the SADC (Shabangu et al., 2022). In addition to desk reviews, there is an emerging dialogue around the concept of mutual recognition and its prospective implementation within the East African Community Medicines Regulatory Harmonisation (EAC-MRH) initiative. Although the EAC is presently focused on work sharing and conducting joint inspections, there is recognised potential for establishing mutual recognition agreements in the future. This discussion is part of a broader movement among Regional Economic Communities (RECs) throughout Africa, which is spearheading regional harmonisation by issuing harmonised regulatory guidelines and setting up Joint Assessment Procedures (JAPs) (Miletic et al., 2023). The three significant regional JAPs are the EAC-MRH, West Africa Medicines Regulatory Harmonisation Project (WA-MRH), and ZaZiBoNa (Ngum et al., 2022; Shabangu et al., 2022; Sithole et al., 2020, p. 2021). These initiatives exemplify the ongoing trend towards fostering regulatory collaboration and harmonisation across the African continent, aiming to streamline regulatory processes and improve access to quality medicines (Miletic et al., 2023; Sithole et al., 2021).

The fiscal year 2019/2020 witnessed a reduction in compliance with GMP requirements among the inspected facilities, with only 32.9% of 76 facilities meeting the necessary standards compared with 50% of 120 facilities in 2018/2019. This decrease may be attributable to various factors, such as an enhanced understanding and expertise of inspectors, particularly in the production field, which has led to more rigorous and detailed assessments. Conversely, this might suggest a lapse in adherence to good production practices by manufacturing facilities during this period. Interestingly, there was a substantial increase in the number of facilities categorised as ‘on demand’ in 2019/2020, accounting for 56.6% of the facilities, compared to only 29.2% in 2018/2019. This shift indicates a growing need for improved manufacturing practices and regulatory compliance in the pharmaceutical sector. Geyer et al. observed similar trends in their 2018 study, which investigated the deficiencies observed by the Brazilian Health Regulatory Agency (ANVISA) on Good Manufacturing Practices International Inspections (Geyer et al., 2018). They found that conformance gaps were prevalent, indicating the crucial need for continuous monitoring and improvement of manufacturing practices. As TMDA continues its mission to ensure the quality, safety, and efficacy of medicines and medical devices, insights from these inspection outcomes can inform future strategies to improve GMP adherence in the industry.

Further analysis revealed significant findings regarding GMP principles. One crucial flagged out aspect was good quality control laboratory practices, in which 16 facilities (48.5%) showed critical non-conformance. This cements the need for tighter quality control measures to ensure that the products meet the required specifications and are safe for patient use (Lee et al., 2015).

Non-conformance with quality control can lead to sub-standard products with inferior therapeutic benefits, posing a health risk to consumers (Stoimenova et al., 2020). Therefore, responsible industries must promptly rectify these issues. The inspection data serves as an invaluable resource for TMDA, helping to identify areas needing more focus and enabling the development of targeted training programmes for inspectors. Such programmes can bolster their expertise, ensuring more effective future inspections and ongoing improvements in conformance levels (Nayyar et al., 2015).

Moreover, the data also emphasise the importance of a tiered classification system for non-conformance (Geyer et al., 2018; Stoimenova et al., 2020). This system enables a targeted approach to problem resolution, ensuring that resources are allocated where they can have the most substantial impact (Geyer et al., 2018). Critical non-conformances, for instance, require immediate attention, given their potential to compromise a product's safety, efficacy, and quality (Stoimenova et al., 2020).

Interestingly, the non-conformance findings were not linked to specific types of pharmaceutical products or formulations. All facilities with critical non-conformances found across both periods were manufacturing general pharmaceutical products. These included formulations such as tablets, capsules, small-volume parenterals, dry powders for injection, suspensions, and external preparations. This broad distribution of non-conformances across different types of products indicates that quality issues and challenges can occur, irrespective of the type of pharmaceutical product being manufactured. Therefore, a practical regulatory framework and stringent GMP practices are essential to ensure quality, safety, and efficacy.

We propose two key policy recommendations based on our findings to boost GMP compliance and strengthen regulatory frameworks in Tanzania and East Africa. First, facilities that have consistently met GMP standards for three consecutive years should qualify for desk reviews, optimise the inspection process, and better allocate regulatory efforts. This incentivizes adherence to GMP standards and focuses on on-site inspections of potentially non-compliant facilities. Second, we advise regional governments to support pharmaceutical manufacturing with targeted training programmes, especially in areas of critical non-compliance identified in our study. Such focused training can bridge knowledge gaps and increase GMP compliance, thereby enhancing the quality of pharmaceutical products across the region.

## Study limitations

Some limitations of this study should be considered when interpreting the findings. First, the study primarily focused on GMP compliance within a selected sample of pharmaceutical industries in a few countries, which may not apply to the global pharmaceutical landscape. On the other hand, this study categorises non-compliance as minor, major, or critical. It does not delve deeply into the underlying causes of non-compliance issues in different countries, which would help pharmaceutical industries learn about possible interventions and improvements. Finally, this study does not comprehensively explore the long-term implications of critical non-compliance on public health, the pharmaceutical industry's reputation, or the broader availability of safe and effective medicines, which is important for understanding the wider impact of non-compliance.

Another limitation stems from the data being based on inspection reports conducted by various inspectors. These inspectors may have differing interpretations of the guidelines or GMP requirements, potentially introducing variability into the evaluation process. This challenge was addressed by standardising the guidelines and inspection tools to harmonise the interpretation and assessment of compliance, thereby striving to ensure a more uniform and objective analysis.

## Conclusion

GMP inspection is essential to maintain the quality of pharmaceutical products. Adopting effective strategies, such as a tiered classification system for non-conformances, can significantly improve the effectiveness of these inspections. The TMDA will continue to conduct such inspections to ensure that good-quality products are always introduced into the Tanzanian market.

## Data Availability

All the data analyzed in this study were included in this article.

## Data access statement

All research data supporting this publication are available in the manuscript.
